# Prevalence, etiology, clinical features and management associated with impacted and transmigrated mandibular canines: a systematic review

**DOI:** 10.1186/s12903-023-03717-1

**Published:** 2023-12-07

**Authors:** Haritha Pottipalli Sathyanarayana, Ludovica Nucci, Fabrizia d’Apuzzo, Letizia Perillo, Sridevi Padmanabhan, Vincenzo Grassia

**Affiliations:** 1 Department of Orthodontics and Dentofacial Orthopaedics, Sri Ramachandra Dental College & Hospital, Sri Ramachandra Institute of Higher Education and Research (SRIHER), No 1, Sri Ramachandra Nagar, Chennai, Tamilnadu 600116 India; 2 Multidisciplinary Department of Medical-Surgical and Dental Specialties, University of Campania Luigi Vanvitelli, via Luigi De Crecchio 6, Naples, 80138 Italy

**Keywords:** Mandibular canine, Impaction, Transmigration, Prevalence, Etiology, Treatment

## Abstract

**Background:**

The occurrence of mandibular canine impaction and/ or transmigration is a rare clinical entity but diagnosis and treatment planning is of clinical significance. The associated etiological factors and the clinical guidelines for the management are still not clear.

The aim of this systematic review was to summarize the available data to report the prevalence and identify the etiological factors, clinical features, and various treatment outcomes in patients with mandibular canine impaction and/or transmigration.

**Methods:**

The review protocol was registered in PROSPERO (CRD42021222566) and was conducted and reported according to the PRISMA and Cochrane Handbook / Preferred Reporting Items for Systematic Reviews and Meta-Analyses statement. A computerized search of studies published up to April 30, 2023, was conducted using the following databases: Medline, Cochrane Database of Systematic Reviews, Cochrane Central Register of Controlled Trials, Scopus, Web of Science, and Latin American and Caribbean Health Sciences Literature. A manual search of the reference and citation lists of eligible articles and existing systematic reviews for any additions were also conducted. The Newcastle–Ottawa Scale quality assessment tool was used to assess the studies' quality.

**Results:**

After removing 6 duplicates, 3700 articles were identified. For the final analysis, 19 studies published between 1985 and 2023 met all the eligibility criteria and were included. A total of 7 studies presented as good and 12 studies presented as satisfactory. Patients were screened in ten studies and diagnostic records from archives were retrieved in nine studies. The total number of diagnostic records screened was 138.394, and the total number of patients from the included studies was 43.127.

**Conclusions:**

Based on the findings from this systematic review, the prevalence of mandibular canine impaction ranged from 0.008% to 1.29% while canine transmigration from 0.12% to 0.98%. Crowding of the mandibular arch, the presence of a retained deciduous canine, and odontoma or cyst are the etiological factors more commonly associated with mandibular canine impaction and or transmigration. Surgical extraction and surgical exposure followed by orthodontic traction are the two most frequently carried out treatment modalities in the management of mandibular canine impaction and or transmigration.

## Background

Canines play an important role in esthetics and functional occlusion. Indeed, they are positioned in the anterior zone of the dentition, and canine-guided occlusion is one of the main goals at the end of orthodontic treatment in permanent dentition. Impaction is defined as the failed eruption of a permanent tooth with a completely developed root [1]. Although there are wide variations among populations as regards the prevalence of impaction, the third molars remain the most prevalent impacted teeth followed by maxillary canines with a prevalence of 1.7% to 4.7% [2–6]. The most commonly reported reasons for maxillary canine impaction are the late eruption between 11 and 12 years following the premolars, and the long root and the position of the adjacent teeth [7–9]. Furthermore, palatally impacted maxillary canines are attributed to hereditary influence, whereas buccally impacted maxillary canines have been attributed to arch length tooth size discrepancies [10–12]. In the lower arch, excluding the third molars, canines are the most frequently impacted teeth followed by the second premolars [13] although it occurs approximately twenty times less than the maxillary canines [8]. Thus, impaction of permanent mandibular canines is a rare developmental disturbance but it represents a clinical concern and challenge to orthodontists. Abnormal displacement of the tooth bud in embryonic life is one of the most commonly accepted etiological factors [14]. Other possible reasons investigated are crowding in the anterior area with lack of space in the dental arch, premature loss or over-retention of the deciduous teeth, excessive canine crown length, hereditary factors, functional disturbances of the endocrine glands, tumors, cysts, and mandibular trauma [15]. Root resorption of the adjacent teeth, the drift of the lateral incisor, and dentigerous cyst formation are the most common complications associated with mandibular canine impaction [15]. A particular manifestation of mandibular canine impaction is transmigration which has a reported prevalence of 0.18%-0.55% [16–18]. Javid and Mupparapu defined transmigration as a condition in which more than 50% of the total length of the canine has crossed the midline [19, 20], whereas Tarsitano et al. considered a canine as transmigrated when the tooth crosses the midline in its pre-eruptive phase [21]. Over-retention of the primary canines, proclination of the incisors, and enlarged symphyseal area are the typical signs of mandibular canine transmigration [22].

Rotation of tooth buds has also been proposed as an etiologic factor for impaction. Therefore, in the presence of a strong eruptive force, mesioangular or horizontal rotation of the tooth bud may result in transmigration unless the tooth faces resistance from tooth roots, neighboring anatomic structures, or dense bone [22]. Peck observed that mandibular canine transmigration was associated with other dental anomalies such as hypodontia, palatally displaced canines, and bilateral occurrence and suggested a possible genetic association among etiological factors [23]. However, although multiple aspects have been described, the etiology and the exact mechanism of mandibular canine impaction and/or transmigration are still unclear [24].

Considering the scarce literature available on this topic, which mainly comprises case reports, case series and few observational and interventional studies, diagnosis and management of mandibular canine impaction remain a challenge to orthodontists [25]. Only one previous systematic review has included 13 studies, published till 2016**,**whose 6 were observational, 2 were cross-sectional, and 5 were cohort studies using panoramic x-rays as diagnostic records. The prevalence reported in that publication showed values from 0.9% to 1.35% [26]. However, against the need for further required clarifications, it is pivotal to deeply investigate this topic in order to better understand the phenomenon of mandibular canine transmigration [27]. The analysis of the results from published studies would allow a better knowledge of the prevalence in various populations, on the etiology and diagnostic approaches with a focus on treatment planning. Therefore, the aim of this systematic review was to summarize the available data to report the prevalence, to identify the main etiological factors, the most common clinical features, and the various treatment approaches in patients with mandibular canine impaction and/or transmigration.

## Methods

### Protocol and registration

The review protocol was made a priori, registered in PROSPERO (CRD42021222566), and conducted, and reported according to the Cochrane Handbook/Preferred Reporting Items for Systematic Reviews and Meta-Analyses statement [28].

### Eligibility criteria

According to the PICO (participants, intervention, comparison, and outcome) design schema: (P) human participants of any age, sex, ethnicity or malocclusion, (I) diagnosed with impacted and/or transmigrated mandibular canine with or without treatment, (C) described in observational and interventional clinical studies (O) assessing their prevalence, etiology, clinical features, and management were included. No limitations concerning language, publication year, or status were applied. Excluded studies were case reports, case series, book chapters, conferences’ abstracts, author debates, summary articles, non-clinical studies and investigations on animals, interviews, and commentaries. The primary outcome of this review was the evaluation of the prevalence of impacted and/or transmigrated mandibular canines among various populations, the etiological factors of the canine impaction and/or transmigration including the associated clinical features, while the secondary outcome was the treatment approaches and outcomes in patients with mandibular canine impaction and/or transmigration.

### Information sources and search strategy

The search strategy was conducted independently by two authors. Six electronic databases (Medline, Cochrane Database of Systematic Reviews, Cochrane Central Register of Controlled Trials, Scopus, Web of Science, Latin American and Caribbean Health Sciences Literature) were searched systematically without restrictions for publication date, language, or type from inception up to April 2023, whereas the Digital Dissertations and Google Scholar, as well as reference and citation lists of eligible articles and existing systematic reviews, were manually searched for any additions. The keywords used for the search were the following: “Cuspid”[All Fields] AND “lower”[All Fields]) OR (“Canine”[All Fields] AND “lower”[All Fields]) OR (“Canine”[All Fields] AND “mandibular”[All Fields]) OR (“Cuspid”[All Fields] AND “mandibular”[All Fields])) AND (“Impaction”[All Fields] OR “ectopic”[All Fields] OR “transmigrated”[All Fields] OR “ectopic”[All Fields] OR “transposed”[All Fields] OR “displaced”[All Fields] OR “malpositioned”[All Fields]).

### Study selection process, data items, and collection process

Two authors screened titles, abstracts, and full texts of identified studies to check for eligibility. Any differences between reviewers were resolved by discussion with a third author. Ambiguities were resolved after consulting the other authors. Data extraction from each report was performed independently without blinding by two reviewers with similar discrepancy resolution using pre-defined and piloted forms covering: (1) study characteristics (design, country, population); (2) patient characteristics (mean age and sex); (3) prevalence in the population; (4) radiological assessment type; (5) etiological factors; (6) treatment approaches. The corresponding authors were contacted when missing information, additional data and/or specific clarifications were required for our assessment.

### Study quality and risk of bias assessment

The modified Newcastle–Ottawa scale was used by the same researchers to assess the quality of the studies (Ottawa). In case of a disagreement between the two initial researchers, a consensus was reached, whereas a third investigator was consulted in case of a question. The risk of bias in individual studies was assessed according to Cochrane guidelines with the Risk of Bias 2.0 tool independently by two authors with the same discrepancy resolution reached through discussion [29].

### Synthesis methods and effect measures

An effort was made to maximize data synthesis; thus, the studies were included if did not completely report all information requested. Non-parametric summary data was converted into parametric data and in case of missing data, data was handled by calculating or converting with the available information or requesting supplemental data from the corresponding authors. For each outcome, the mean difference was preferably used as effect measures in the presentation of results. Data extraction forms were subsequently compared between the researchers and a final version was constructed upon discussion and agreement.

## Results

### Study selection and flow diagram

**Fig. 1:**
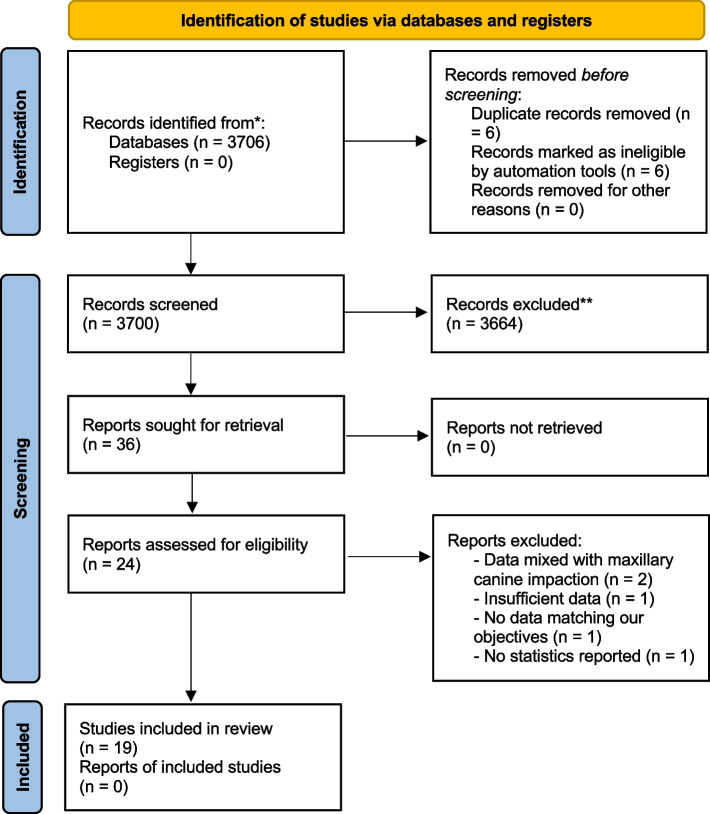
PRISMA flow chart

The initial search identified a total of 3706 articles. After removing 6 duplicates, 3700 articles were screened and 3664 articles were excluded after reading the title and abstract. 12 articles were eliminated after reading the complete full text. The remaining 24 articles were analyzed. Among these, 5 were excluded for these reasons: 2 studies reported combined data with maxillary canine impaction, 1 study presented insufficient data, 1 study reported data not matching our objectives and 1 study did not provide statistical data. In the end, 19 articles were included in the qualitative synthesis (Fig. 1).

### Study characteristics

Out of the 19 studies, 3 were case–control studies (2 prospectives [30, 31] and 1 retrospective [27]) and 16 were cross-sectional studies (2 prospectives [16, 18] and 14 retrospectives [6, 30, 32–43]). Thus, a total number of 43.127 patients were prospectively screened, whereas 138.394 diagnostic records were retrieved retrospectively from archives.

The patients’ characteristics in the selected studies included the presence of impacted and/or transmigrated mandibular canines in permanent dentition.

### Risk of bias in studies

The Risk of Bias was assessed using the modified Newcastle–Ottawa scale (Table 1). The 19 studies included in this systematic review showed varying levels of quality [24]. A total of 7 studies were rated as ‘good’ [6, 18, 27, 32, 33, 40, 44], whereas 12 studies as ‘satisfactory' [16, 30, 31, 34–39, 41–43]. Two studies showed a true representative population [30, 37] Most of the study samples were somewhat representative of the population or had convenient samples. Three studies had a comparable control group [27, 34, 44]and eleven studies reported complete statistical data with relevant analysis [6, 18, 27, 30, 32, 33, 36, 38–40, 44].

**Table 1:** Studies’ quality assessment according to the Newcastle–Ottawa Scale

First author (year)	Selection: Representativeness of the sample	Selection: Sample size	Selection: non-respondents	Selection: ascertainment of the exposure	Comparability	Outcome: assessment of the outcome	Outcome: statistical test	Score	Quality of the study
Al-Abdadallah (2018) [32]	b. Somewhat representative of the average in the target population*	b. Not justified	Not applicable	a. validated measurement tool **	b. the study control for any additional factor *	b. Record linkage **	a. the statistical test used to analyse the data is clearly described and appropriate *	7	good
Aktan (2010) [6]	b. Somewhat representative of the average in the target population*	b. Not justified	Not applicable	a. validated measurement tool **	b. the study control for any additional factor *	b. Record linkage **	a. the statistical test used to analyse the data is clearly described and appropriate *	7	good
Aydin (2004) [16]	b. Somewhat representative of the average in the target population*	b. Not justified	Not applicable	a. validated measurement tool **	b. the study control for any additional factor *	b. Record linkage **	b. statistical test not appropriate, not described or incomplete	5	satisfactory
Azeem (2019) [33]	b. Somewhat representative of the average in the target population*	b. Not justified	Not applicable	a. validated measurement tool **	b. the study control for any additional factor *	b. Record linkage **	a. the statistical test used to analyse the data is clearly described and appropriate *	7	good
Bertl (2019) [34]	c. Convenient sample	b. Not justified	Not applicable	a. validated measurement tool **	a. the study controls for the most important factor*	b. Record linkage **	b. statistical test not appropriate, not described or incomplete	5	satisfactory
Buyukkurt (2007) [35]	b. Somewhat representative of the average in the target population*	b. Not justified	Not applicable	a. validated measurement tool **	b. the study control for any additional factor *	b. Record linkage **	b. statistical test not appropriate, not described or incomplete	6	satisfactory
Celikoglu (2010) [30]	c. Convenient sample	b. Not justified	Not applicable	a. validated measurement tool **	b. the study control for any additional factor *	b. Record linkage **	a. the statistical test used to analyse the data is clearly described and appropriate *	6	satisfactory
Jain (2015) [44]	a. Truly representative of the average in the target population*	b. Not justified	Not applicable	a. validated measurement tool **	a. the study controls for the most important factor*	b. Record linkage **	a. the statistical test used to analyse the data is clearly described and appropriate *	7	good
Jain (2019) [31]	c. Convenient sample	b. Not justified	Not applicable	a. validated measurement tool **	b. the study control for any additional factor *	b. Record linkage **	b. statistical test not appropriate, not described or incomplete	5	satisfactory
Kamiloglu (2014) [36]	c. Convenient sample	b. Not justified	Not applicable	a. validated measurement tool **	b. the study control for any additional factor *	b. Record linkage **	a. the statistical test used to analyse the data is clearly described and appropriate *	6	satisfactory
Kara (2011) [37]	a. Truly representative of the average in the target population*	b. Not justified	Not applicable	a. validated measurement tool **	b. the study control for any additional factor *	b. Record linkage **	b. statistical test not appropriate, not described or incomplete	6	satisfactory
Karabas (2020) [38]	c. Convenient sample	b. Not justified	Not applicable	a. validated measurement tool **	b. the study control for any additional factor *	b. Record linkage **	a. the statistical test used to analyse the data is clearly described and appropriate *	6	satisfactory
Koç (2021) [39]	c. Convenient sample	b. Not justified	Not applicable	a. validated measurement tool **	a. the study controls for the most important factor*	b. Record linkage **	a. the statistical test used to analyse the data is clearly described and appropriate *	6	satisfactory
Marra (2021) [40]	b. Somewhat representative of the average in the target population*	b. Not justified	Not applicable	a. validated measurement tool **	b. the study control for any additional factor *	b. Record linkage **	a. the statistical test used to analyse the data is clearly described and appropriate *	7	good
Plakwicz (2019) [27]	b. Somewhat representative of the average in the target population*	b. Not justified	Not applicable	a. validated measurement tool **	a. the study controls for the most important factor*	b. Record linkage **	a. the statistical test used to analyse the data is clearly described and appropriate *	7	good
Sajnani (2012) [41]	c. Convenient sample	b. Not justified	Not applicable	a. validated measurement tool **	b. the study control for any additional factor *	b. Record linkage **	b. statistical test not appropriate, not described or incomplete	5	satisfactory
Sharma (2014) [18]	b. Somewhat representative of the average in the target population*	b. Not justified	Not applicable	a. validated measurement tool **	b. the study control for any additional factor *	b. Record linkage **	a. the statistical test used to analyse the data is clearly described and appropriate *	7	good
Stabryla (2021) [42]	b. Somewhat representative of the average in the target population*	b. Not justified	Not applicable	a. validated measurement tool **	b. the study control for any additional factor *	b. Record linkage **	b. statistical test not appropriate, not described or incomplete	6	satisfactory
Yavuz (2007) [43]	c. Convenient sample	b. Not justified	Not applicable	a. validated measurement tool **	b. the study control for any additional factor *	b. Record linkage **	b. statistical test not appropriate, not described or incomplete	5	satisfactory

^*^ and ^**^ correspond to ratings assigned for each item according to The Newcastle-Ottawa Quality Assessment Scale (https://www.ohri.ca/programs/clinical_epidemiology/oxford.asp)

**Fig. 2:**
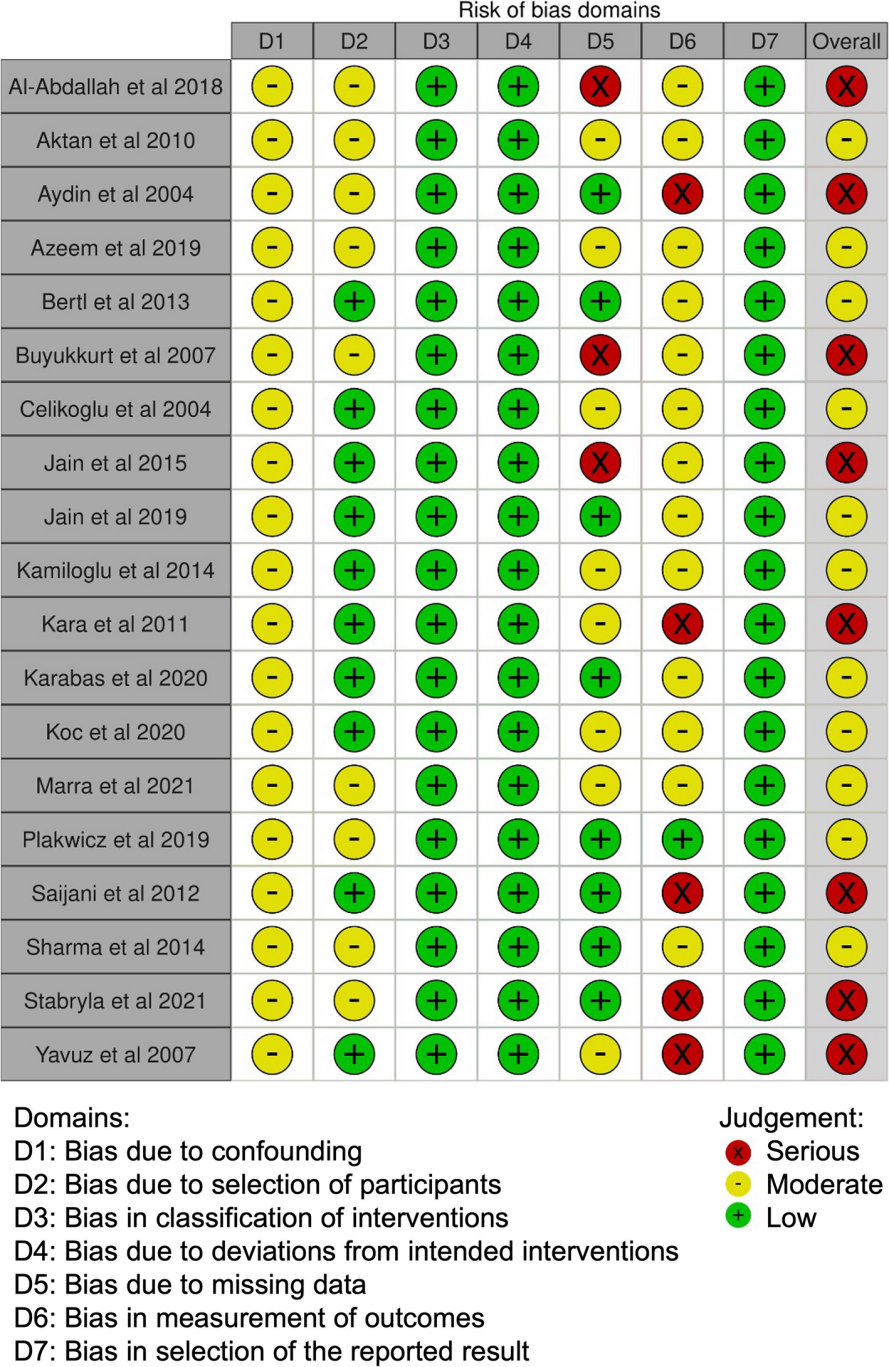
Risk of bias of included studies

**Fig. 3:**
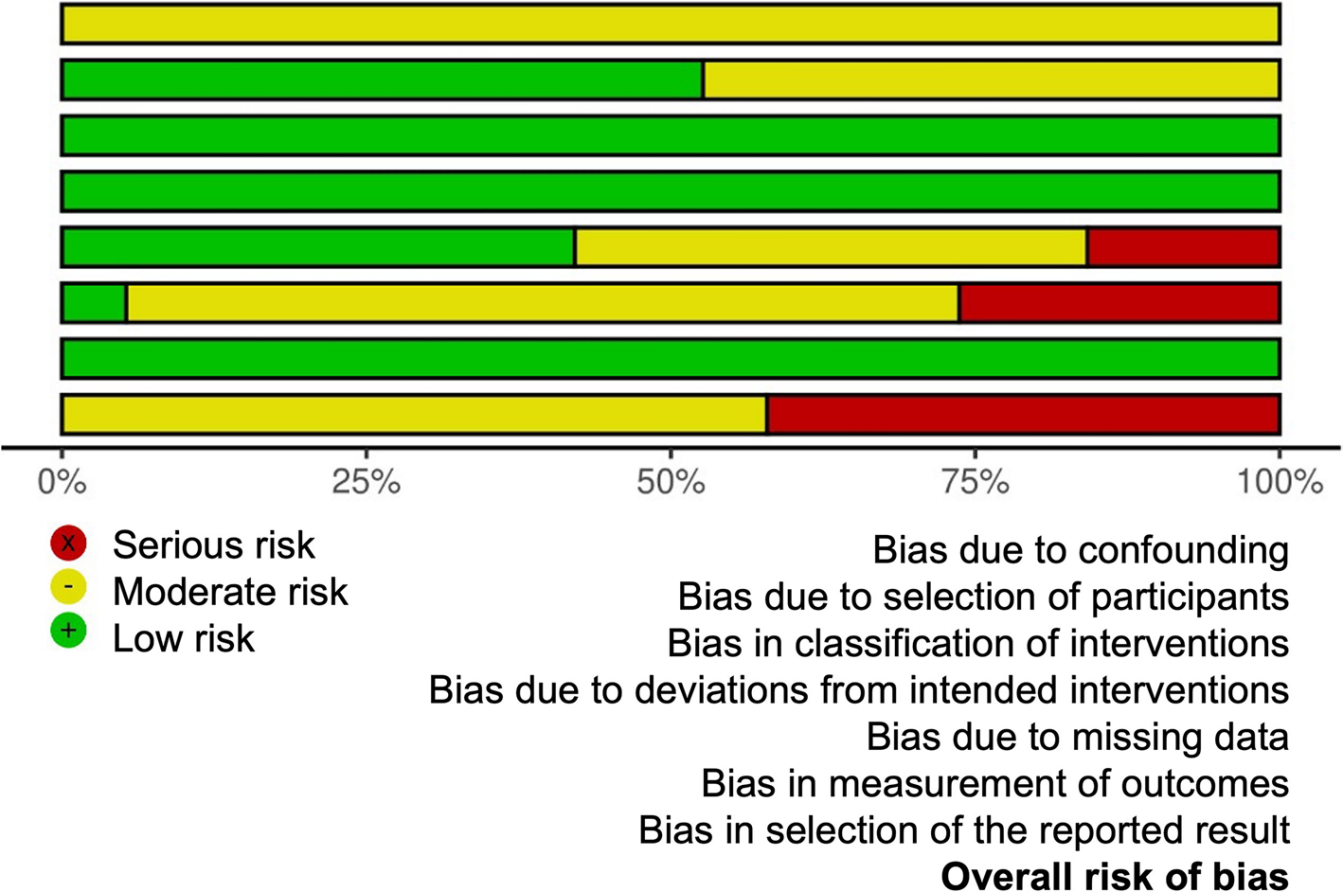
Summary plot of risk of bias

The risk of bias in the individual studies was assessed through the last version of the (ROBINS-E) (https://www.riskofbias.info/welcome/robins-e-tool) and it is presented in Figs. 2 and 3.

### Results of individual studies

The majority of the studies were retrospective and the records were screened and analyzed and depicted in Table 2. Bertl et al. [34] reported the maximum study samples of 94 patients followed by Plakwicz et al. [27] with 93 patients. Included studies assessed populations from the following countries: Jordan [32], Turkey [6, 16, 30, 34, 37, 39, 43], Pakistan [33], Austria [34], India [18, 31, 44], Northern Cyprus [36], Italy [40], Poland [27, 42], and Southern Chinese [41]. Information on gender was provided in 14 studies out of 19 [6, 16, 30, 33–36, 38, 40–44]. The Orthopantomogram (OPG) was used as a diagnostic radiograph in 14 studies [6, 16, 18, 27, 30–33, 35–37, 41, 43, 44], CBCT in 3 studies [34, 38, 39], and both OPG and CBCT in only 2 studies [40, 42]. The number of reported impacted mandibular canines from the included studies was 802. A total of 580 patients were reported from 14 studies and the total number of impacted canines from these studies was 620. Five studies reported data with only the number of impacted canines as 182 [18, 27, 31, 39, 42].

**Table 2:** Studies involved in qualitative analysis reporting the sample and prevalence of mandibular canine impaction and / transmigration

First author (year)	Journal	Study design	Population sample	Screened records or patients	Patients with impaction	Mean age years (range)	Gender (%)		Impacted/transmigrated mandibular canines			Prevalence (%)		Radiological assessment
							**M**	**F**	**unilateral**	**bilateral**	**tot n°**	**impacted**	**transmigrated**	
Al-Abdadallah (2018) [32]	BMC Oral Health	retrospective	Jordan	2979 records	24	23.4 (15 to 40)	46.6	53.4	24	0	24	0.8%	NA	OPG
Aktan (2010) [6]	Eur J Orthod	retrospective	Turkish	5000 records	40	34 (16 to 76)	27.5	72.5	40	3	46	0.46%	0.34%	OPG
Aydin (2004) [16]	Dentomaxillofac Radiol	prospective	Turkish	4500 patients	28	31.8 (NA)	46.4	53.6	26	2	30	0.44%	0.18%	OPG
Azeem (2019) [33]	Dent Press J Orthod	retrospective	Asian / Pakistan	2550 patients	25	23.1 ± 4.1 (16–30)	36	64	25	0	25	NA	0.98%	OPG
Bertl (2019) [34]	Clin Oral Investig	retrospective	Austrian	88 patients	88	25.6 (9.5 to 64.4)	46.6	53.4	88	6	94	NA	NA	CBCT
Buyukkurt (2007) [35]	J Oral Maxillofac Surg	retrospective	Turkish	4500 records	15	25 (13 to 57)	60	40	15	0	15	NA	0.33%	OPG
Celikoglu (2010) [30]	J Oral Maxillofac Surg	retrospective	Turkish	2215 records	14	19.17 (16 to 25)	28.6	71.4	14	0	14	0.4%	0.22%	OPG
Jain (2015) [44]	Aust Orthod J	prospective	Indian	10,422 patients	48	15.03 ± 0.49 (14 to 16)	33.3	66.7	41	7	55	0.4%	NA	OPG
Jain (2019) [31]	Med Pharm Rep	prospective	Indian	1593 patients	NA	NA	NA	NA	NA	NA	8	0.37%	0.12%	OPG
Kamiloglu (2014) [36]	BMC research notes	retrospective	Northern Cypriot	453 patients	4	NA	44.4	55.6	4	0	4	.008%	NA	OPG
Kara (2011) [37]	Med Oral Pat Oral Cir Bucal	retrospective	Turkish	112,873 records	82	38.5 (NA)	NA	NA	82	NA	NA	NA	0.8%	OPG
Karabas (2020) [38]	Oral Radiol	retrospective	Turkish	2591 records	58	26 (12 to 65)	51.7	48.3	50	8	66	.01%	.011%	CBCT
Koç (2021) [39]	Oral Maxillofacial Surg	retrospective	Turkish	2901 records	NA	24.74 (14 to 67)	NA	NA	NA	NA	30	NA	0.55%	CBCT
Marra (2021) [40]	Eur J Paed Dent	retrospective	Italian	200 / 92 records	26	NA (9 to 14)	57.7	42.3	26	2	30	8.9%	1.4%	OPG / CBCT
Plakwicz (2019) [27]	Eur J Orthod	retrospective	Polish	93 records	NA	14.4 (7.6 to 49.5)	NA	NA	93	NA	93	NA	NA	OPG
Sajnani (2012) [41]	J Child Dental	retrospective	Southern Chinese	20,347 patients	63	14.2 ± 3.1 (NA)	49.2	50.8	62	1	64	0.3%	NA	OPG
Sharma (2014) [18]	Int Sch Res Notices	prospective	Indian	3000 patients	NA	24.1 (15 to 53)	NA	NA	15	NA	15	NA	0.5%	OPG
Stabryla (2021) [42]	J Am Dental Assoc	retrospective	Polish	102 patients	NA	15.2 ± 9.3 (7.5 to 53.9)	40.6	59.4	32	2	36	0.35%	NA	OPG or CBCT
Yavuz (2007) [43]	J Contemp Dent Pract	retrospective	Turkish	5022 patients	65	25.3 (NA)	49.2	50.8	65	3	71	1.29%	NA	OPG

### Prevalence

The prevalence of mandibular canine impaction and transmigration varies with the population studied and is depicted in Table 2. The prevalence of mandibular canine impaction and transmigration reported from various studies ranged from 0.008%-1.29% and 0.12%-0.98%, respectively. Considering the mean age at the time of reporting from 17 studies, the lowest mean age was 14.2 years and the highest mean age was 38.5 years. Considering the average gender distribution, mandibular canine impaction and/or transmigration were reported in 44.6% of males and 55.4% of females. The distribution between unilateral and bilateral impaction is also reported in Table 2.

### Etiology

Etiological factors associated with mandibular canine impaction and/or transmigration were reported in 10 studies (Table 3) [6, 18, 27, 34, 35, 37, 38, 41, 43, 44]. The presence of retained deciduous canine, odontoma or cyst, presence of crowding in the mandibular arch, history of trauma to the mandible, and mandibular lateral incisor anomalies were the possible etiological factors noted. The most commonly attributed etiological factor was crowding (6.7%-74.5%), followed by the presence of retained deciduous canine (4.8%-61.3%) and the presence of odontoma or cyst (8.5%-29.41%). Among 19 studies, mandibular lateral incisor anomaly (5.3%) was assessed only by Bertl et al. [34], and trauma to the mandible (1.4%) was reported only by Yavuz et al. [43] and Mupparappu’s classification [20] to classify the pattern of transmigration was used in 9 studies [18, 27, 30, 33–38] to describe the type of mandibular canine transmigration. Type 1 was the most common type of occurrence (11%-76%) followed by Type 2 (12%—45.7%) and the least common was Type 4 (0 canines, 13.3%) and Type 5 (0 canines, 12.5%). The canine crown position was assessed in 4 studies [34, 38, 41, 42]. The most common position was buccal (55.3%-66.7%) and central within the alveolus (53%) in the study by Karabas et al. [38]. The lingual impaction was the least common of all the three positions.

**Table 3:** Studies reporting the etiological factors and clinical features of impacted and / transmigrated mandibular canines

First author (year)	Total n° impacted/ transmigrated mdb canines	Presence of Retained Deciduous Canine (n, %)	Odontome / Cyst (n, %)	Crowding (n, %)	Trauma to mandible (%)	Mdb Lateral Incisor anomalies (%)	Location by Mupparapu classification type (n, %)	Crown position / inclination (n, %)
Aktan (2010) [6]	20	8, 41.2%	5, 29.41%	NA	NA	NA	NA	NA
Azeem (2019) [33]	25	NA	NA	NA	NA	NA	Type 1: 19, 76% Type 2: 3, 12% Type 5: 3, 12%	NA
Bertl (2019) [34]	94	49, 52.1%	17, 18.1%	NA	NA	5.3%	Type 1: 21, 55.3% Type 2: 9, 23.7% Type 3: 4, 10.5% Type 5: 4, 10.5%	Labial: 55.3% Central: 36.2% Lingual: 8.5%
Buyukkurt (2007) [35]	15	6, 40%	2, 13.3%	NA	NA	NA	Type 1: 5, 33.3% Type 2: 3, 20% Type 3: 4, 26.7% Type 4: 1, 6.7% Type 5: 2, 12.5%	NA
Celikoglu (2010) [30]	14	NA	NA	NA	NA	NA	Type 1: 3 Type 2: 2	NA
Jain (2015) [44]	55	NA	NA	Mild: 11, 22.9% Moderate: 16, 33.3% Severe: 14, 29.2%	NA	NA	NA	NA
Kamiloglu (2014) [36]	4	NA	NA	NA	NA	NA	Type 1–1, 25% Type 2–1, 25%	NA
Kara (2011) [37]	82	45.5%	7, 8.5%	NA	NA	NA	Type 1: 9, 11% Type 2: 10, 12.2%	NA
Karabas (2020) [38]	66	38, 57.6%	12, 18.2%	NA	NA	NA	Type 1: 6, 17.1% Type 2: 16, 45.7% Type 3: 4, 11.4% Type 4: 4, 11.4% Type 5: 1, 2.8%	Labial: 16, 24.2% Central: 35, 53.03 Lingual: 7, 10.6 Oblique: 8, 12.12
Plakwicz (2019) [27]	93	57, 61.3%	NA	36, 38.7%	NA	NA	Type 1: 60, 64.5% Type 2: 22, 23.7% Type 3: 5, 5.4% Type 4: 4, 4.3% Type 5: 2, 2.2%	NA
Sajnani (2012) [41]	63	3, 4.8%	NA	NA	NA	NA	NA	Buccal: 40, 63.5% Lingual: 16, 25.4% Central: 6, 9.5%
Sharma (2014) [18]	15	2, 13.3%	2, 13.3%	1, 6.7%	NA	NA	Type 1: 10, 67% Type 2: 2, 13.3% Type 4: 2, 13.3% Type 5: 1, 6.7%	NA
Stabryla (2021) [42]	36	NA	NA	NA	NA	NA	NA	Labial: 24, 66.7% Lingual: 5, 13.9% Central:7, 19.4%
Yavuz (2007) [43]	71	12, 18.5%	6, 11.2%	NA	1, 1.4%	NA	NA	NA

*NA* Not applicable

### Treatment outcomes

Three studies assessed the treatment outcomes of 122 impacted and/or transmigrant mandibular canines (Table 4) [35, 42, 43]. Of these three, Buyukkurt et al. [35] reported that out of 15 impacted canines, 12 were extracted (80%) and the outcome of 3 impacted canines was not mentioned. Thus, we were able to summarize various treatment approaches and outcomes for 119 impacted mandibular canines reported from the above three studies. Observation without any treatment (18 canines, 14.7%), space opening through orthodontic treatment without surgical procedure (3 canines, 2.4%), surgical exposure of the impacted canine followed by orthodontic traction (31 canines, 25.4%), autotransplantation (10 canines, 8.2%) and extraction (57 canines, 46.7%). The most common treatment modality was extraction in two studies and autotransplantation in only one study. Considering the outcomes, we surmise that the position of 34 impacted canines was favorable (28.6%) and 85 canines were unfavorable (71.4%).

**Table 4:** Studies reporting the treatment approaches and outcomes for impacted and / transmigrated mandibular canines

First author (year)	Total impacted and / transmigrated mandibular canines		Observation no treatment (n, %)	Interceptive Treatment / Space opening with orthodontics (n, %)	Surgical canine exposure followed by orthodontics (n, %)	Autotrans plantation (n, %)	Mandibular canine extraction (n, %)
	**patients**	**canines**					
Buyukkurt (2007) [35]	15	15	NA	NA	NA	NA	12, 80%
Stabryla (2021) [42]	32	36	12, 33.3%	3, 8.3%	8, 22.2%	9, 25%	4, 11.1%
Yavuz (2007) [43]	65	71	6, 8.5%	NA	23, 32.4%	1, 1.4%	41, 57.7%

*NA* Not applicable

Stabryla et al. [42] reported two main treatment procedures, the trans alveolar transplantation (9 canines), with a success rate of 89%, and the surgical exposure with orthodontic traction (8 canines), whereas the least common approach, was the space opening without surgery followed by observation. Yavuz et al. [43] reported that among 71 impacted mandibular canines, extraction was the most common procedure (57.7%) followed by surgical exposure and orthodontic traction (32.4%), observation (8.4%) and autotransplantation (1.4%).

## Discussion

The purpose of this systematic review of the literature was to report the prevalence, etiological factors, clinical features, and treatment outcomes in patients with mandibular canine impaction and/or transmigration. The populations mainly assessed in literature for impacted and/or transmigrated mandibular canines were from Turkey and India, whereas only a few samples were selected from other European countries. There were no studies from the American or African continents, so the data lacks a worldwide prevalence. The mean age of reporting showed a wide range from adolescence to adulthood. This may be due to the usual beginning of an orthodontic evaluation with the request of panoramic x-ray when the young patients show a full permanent dentition while the highest mean age reported may be due to the absence of pain or other problems directly related to a mandibular canine impaction which is accidentally found on diagnostic records. No statistically significant gender disparity was observed in this systematic review, but still, a higher number of females than males were reported, which was in accordance with previous studies on maxillary canine impaction [9]. OPG with clinical findings was used to diagnose canine impaction in most of the studies but the three-dimensional position and the possible contact with adjacent tooth roots or crowns may only be assessed with CBCT [9]. Thus, there has been an increased usage of CBCT as a diagnostic record over recent years for accurate diagnosis and treatment planning. The previous systematic review by Dalessandri et al. [26] included thirteen studies with evaluation performed with only OPGs, whereas in this paper 19 articles were included with either OPG, CBCT, or both. CBCT was used as a diagnostic aid in five studies. From the included studies, the prevalence reported for mandibular canine impaction and transmigration, in a representative sample of the populations assessed, was from 0.008% to 1.29% and transmigration was from 0.12%—0.98%. Marra et al. [40] reported a prevalence of mandibular canine impaction of 8.9% and 1.4% of transmigration which were higher than other studies and this was not included in the range represented by other studies because it seemed like an outlier. The probable reason could be that the sample was selected from the archives of the Maxillofacial and Oral Unit of the hospital recruiting a higher number of patients with craniofacial anomalies referred from private practices. Most of the included studies have been on patients reporting to institutions for treatment, hence the prevalence rate reported might not be a true representative of the entire population. Patients with missing canines are more likely to visit for orthodontic consultation compared to patients with normal occlusion.

Among the etiological factors reported in the included studies, the main factors attributed to mandibular canine impaction and/or transmigration were crowding in the anterior area of the lower dental arch and the retention of the deciduous canine over the physiological period of time. The percentage range was not homogeneous in the different samples, maybe due to the different ages of the population assessed. Identifying the etiological factors is important in treatment planning and preventing the tendency for impaction at an earlier stage and subsequent complications at a later stage. Genetic factors are associated with palatally impacted maxillary canines [10]. No study thus far has evaluated the genes associated with mandibular impacted canines and transmigrated canines. For canine transmigration, Type 1 of Mupparapu’s classification was the most common presentation with the buccal position. This data may be strictly related to the main etiological factor, which is the lack of space in the correct position in the lower dental arch that led the tooth to be slightly displaced anteriorly and mesially where there is more symphysial space.

The strength and importance of this systematic review is the update of the available information reported in Dalessandri et al. about the prevalence, and etiology of mandibular impacted canines and transmigrated canines dated till 2016, only evaluated on bi-dimensional diagnostic records. More data from three-dimensional diagnostic records were assessed allowing a better evaluation of the canine crown and root position and its relationship to its neighboring structures. Furthermore, studies describing the therapeutic approaches were also checked as a secondary objective. Only three studies described the clinical approach of managing mandibular canine impaction and the majority of clinicians seem to choose the observation over time through diagnostic OPGs or resorting to extractions during adulthood. More complex treatment approaches are reported more commonly as case reports [40] rather than being evaluated as part of a clinical study. A recent systematic review of orthodontic surgical management of impacted mandibular canines reported that good alignment was achieved in most of the cases. In a few cases, bony ridge resorption and gingival recession were observed. The authors also suggested that radiological diagnosis in late mixed dentition remains an important predictive tool [25]. Generally mandibular canine impaction is considered less favorable for alignment compared to its maxillary counterpart perhaps due to the frequent occurrence of transmigration. Our review showed that the percentage of favorable to unfavorable impaction was 28.6% and 71.4% respectively, however, no study or systematic review reported this data for maxillary canines allowing a direct comparison.

A systematic review by Elangovan et al. [45] reported various interceptive treatment approaches such as rapid palatal expansion, headgear, and distalization reduce the chances of impaction and allows unaided eruption of the maxillary canine.

The change in angulation from a vertical to a more horizontal position makes mandibular canines more complex. However, with early diagnosis, interception at the right time, and surgical exposure with orthodontic intervention, impacted mandibular canines can be guided to an appropriate position in the dental arch. Extraction of deciduous canine between 10–13 years of age allows the permanent canine to erupt spontaneously. To date, studies evaluating the various interceptive treatment approaches for the management of potentially impacted mandibular canines are lacking. This study showed that the time of reporting for an orthodontic consultation is rather late. Thus, as a clinical suggestion, to avoid the impaction of the mandibular canine due to lower anterior crowding or any other reason, a first visit during the early mixed dentition to guide a favorable eruption should be mandatorily suggested by general dentists and pediatricians. Early treatment with serial extraction and periodic follow-up may eliminate the occurrence or severity of mandibular impaction and /or transmigration.

It is not clear the reason why there is limited knowledge of this dental positional anomaly in other geographic areas worldwide. Thus, it may be interesting for future research to develop an online survey and epidemiological cross-sectional studies in order to update the prevalence of mandibular canine impaction/transmigration. Subsequently, future studies should focus on the development of a classification system and treatment protocol using first panoramic x-rays and then CBCT evaluations due to the lack of scientific evidence and clinical community agreement [46].

### Limitations

A meta-analysis was not feasible due to the lack of measurable and homogeneous data. Moreover, the majority of the assessed data was retrospective, thus future prospective longitudinal studies should enlighten the various etiological factors in a follow-up evaluation.

## Conclusions

According to the 19 studies included in this systematic review, the mandibular canine impaction showed a prevalence ranging from 0.008% to 1.29% while mandibular canine transmigration a value from 0.12% to 0.98%. The main etiological factors attributed to mandibular canine impaction and/or transmigration are crowding of the mandibular arch, presence of retained deciduous canine, or odontome/cyst. Based on Mupparappu’s classification of transmigration, Type 1 was the most common while Type 5 had the least common occurrence. The most frequent position of the impacted mandibular canine was the buccal impaction, whereas the least common were the lingually impacted canines. Treatment approaches for mandibular canine impaction and/or transmigration were mainly tooth extraction, and surgical exposure followed by orthodontic traction in the arch by fixed appliances*.* Future research studies investigating the updated prevalence, etiology, and management of this clinical issue are warranted.

## Data Availability

The datasets used and/or analysed during the current study are available from the corresponding author on reasonable request.
